# Observation of a photoinduced, resonant tunneling effect in a carbon nanotube–silicon heterojunction

**DOI:** 10.3762/bjnano.6.71

**Published:** 2015-03-10

**Authors:** Carla Aramo, Antonio Ambrosio, Michelangelo Ambrosio, Maurizio Boscardin, Paola Castrucci, Michele Crivellari, Marco Cilmo, Maurizio De Crescenzi, Francesco De Nicola, Emanuele Fiandrini, Valentina Grossi, Pasqualino Maddalena, Maurizio Passacantando, Sandro Santucci, Manuela Scarselli, Antonio Valentini

**Affiliations:** 1 INFN, Sezione di Napoli, Via Cintia 2, 80126 Napoli, Italy; 2 CNR-SPIN U.O.S. di Napoli and Dipartimento di Scienze Fisiche, Università degli Studi di Napoli “Federico II”, Via Cintia 2, 80126 Napoli, Italy; 3 Centro per Materiali e i Microsistemi Fondazione Bruno Kessler (FBK), Trento, Via Sommarive 18, 38123 Povo di Trento, Italy; 4 INFN, Sezione di Roma “Tor Vergata”, Dipartimento di Fisica, Università degli Studi di Roma “Tor Vergata”, Via della Ricerca Scientifica 1, 00133 Roma, Italy; 5 INFN, Sezione di Perugia, Dipartimento di Fisica, Università degli Studi di Perugia, Piazza Università 1, 06100 Perugia, Italy; 6 INFN, Sezione di L’Aquila, Dipartimento di Scienze Fisiche e Chimiche, Università degli Studi dell’Aquila, Via Vetoio, 67100 Coppito, L’Aquila, Italy; 7 INFN, Sezione di Napoli and Dipartimento di Scienze Fisiche, Università degli Studi di Napoli “Federico II”, Via Cintia 2, 80126 Napoli, Italy,; 8 INFN, Sezione di Bari and Dipartimento di Fisica, Università degli Studi di Bari, Via Amendola 173, 70126 Bari, Italy

**Keywords:** heterojunction, multiwall carbon nanotubes, NDR, photodetector, tunneling

## Abstract

A significant resonant tunneling effect has been observed under the 2.4 V junction threshold in a large area, carbon nanotube–silicon (CNT–Si) heterojunction obtained by growing a continuous layer of multiwall carbon nanotubes on an n-doped silicon substrate. The multiwall carbon nanostructures were grown by a chemical vapor deposition (CVD) technique on a 60 nm thick, silicon nitride layer, deposited on an n-type Si substrate. The heterojunction characteristics were intensively studied on different substrates, resulting in high photoresponsivity with a large reverse photocurrent plateau. In this paper, we report on the photoresponsivity characteristics of the device, the heterojunction threshold and the tunnel-like effect observed as a function of applied voltage and excitation wavelength. The experiments are performed in the near-ultraviolet to near-infrared wavelength range. The high conversion efficiency of light radiation into photoelectrons observed with the presented layout allows the device to be used as a large area photodetector with very low, intrinsic dark current and noise.

## Introduction

Negative differential resistance (NDR), where the current decreases as a function of voltage, has been observed in the current–voltage curves of several types of structures (e.g., heavily doped p–n junction, double and triple barrier, quantum well, quantum wires and quantum dots, nanotubes and graphene) [1–7]. In general, it has been associated with the occurrence of a process at the junction that allows the electrons to tunnel between energy levels that are aligned only at a certain applied voltage. In the case of carbon nanotubes (CNTs), a number of cases have been reported in which this effect has been observed both for single-walled as well as for double-walled CNTs [3–5].

In this work, a photosensitive junction was fabricated which exhibits a current–voltage characteristic showing a marked tunneling-like shape with a NDR in the region between 1.5 and 2.2 V of excitation light. In fact, in this region, the observed current decreases and varies with the incident photon wavelength. The effect of the incident radiation is so strong it allows the carriers to cross the junction through the 2.4 V barrier, even at voltages of a few hundred mV.

The optoelectronic properties of semiconducting carbon nanotubes are advantageous for the development of photodetector devices in the near-to-mid-infrared region (from ≈1 to ≈15 μm) [8]. The mechanisms behind the infrared sensitivity of CNTs have been discussed by various authors [9–10]. The photoconductivity of individual CNTs, as well as ropes and films of CNTs have been studied extensively both in the visible [11] and the infrared [12] range. The variations in the photoconductivity of CNT-based devices have been attributed to the photon-induced generation of charge carriers in single-wall CNTs and the subsequent charge separation across the carbon nanotube–metal contact interface [11]. To the best of our knowledge, there is a lack of measurements in the UV region [8], and moreover, there are no reports on the observation of the NDR generated by light radiation to date.

In this paper, we report on the device characteristics, optoelectronic properties and, for the first time, a portion of the I–V curve showing a bell-shape tunneling behavior with a marked presence of a NDR. The tunneling current is generated by the incident radiation and it is function of the wavelength and the incident power intensity.

## Experimental

**Figure 1:**
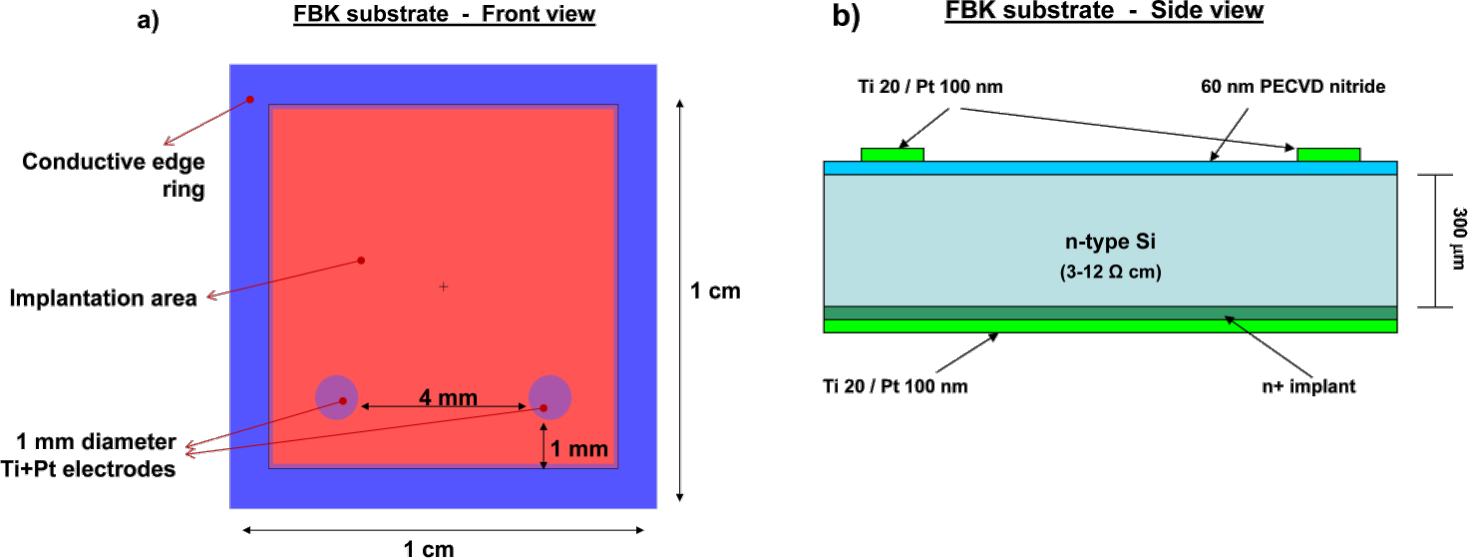
(a) Schematic front view and (b) side view of the Si substrate produced by Fondazione Bruno Kessler (FBK) in Povo, Trento (Italy).

In a similar manner to that described [13], the photodevice was realized by growing a film of multiwall carbon nanotubes (MWCNTs) on an n-doped silicon substrate. The substrates used to build the photodetector were fabricated by Fondazione Bruno Kessler (FBK) in Povo, Trento (Italy), unlike the substrate of the devices shown in Figure 1 of [13]. On the upper part of the n*-*doped silicon wafer (1 × 1 cm^2^, 300 μm thickness and resistivity of 3–12 Ω∙cm) an insulating layer of 60 nm of silicon nitride (Si_3_N_4_) is grown by plasma-enhanced chemical vapor deposition (PECVD). Two, circular, metallic Ti/Pt electrodes of 1 mm in diameter are placed at a distance of 4 mm from each other (Figure 1a) on the silicon nitride surface. A metallic guard ring, 1 mm wide, serves to inhibit superficial current dispersions during electrical measurements. In the bottom part of the silicon wafer, a thin n*^+^* implanted layer ensures ohmic contact between the silicon and the metallic Ti/Pt electrodes, covering the entire back surface (Figure 1b). Thus, the main differences between the FBK substrate used in this work and the substrate used in [13] are: the Si_3_N_4_ insulation layer on the upper part of the Si is much thinner (60 nm instead of 140 nm), the different thickness of Si (300 μm instead of 500 μm), the different Si resistivity (3–12 Ω∙cm instead of 40 Ω∙cm) and the absence of a Si_3_N_4_ insulating layer in the bottom part of the Si layer. Due to these differences, the results from this work are different from those obtained in earlier work reported in [13].

**Figure 2:**
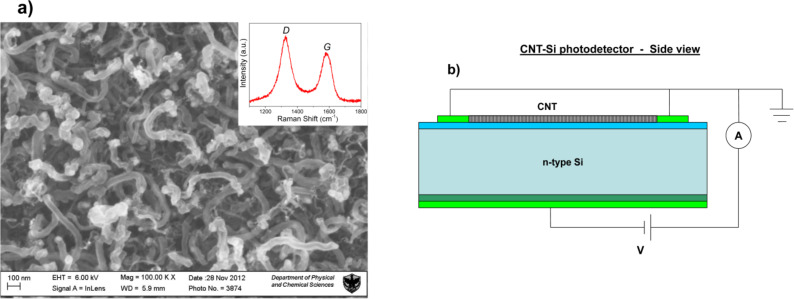
(a) Scanning electron microscopy (SEM) image of MWCNT samples grown on the implantation area. The inset shows a Raman spectrum of the same sample (b) Side view of electrical readout connections.

The FBK substrate was then covered with a uniform layer of MWCNTs grown on the implantation area by CVD. The MWCNTs grow due to the presence of catalytic particles of about 60 nm in diameter, which are obtained by annealing a 3 nm thick Ni film at 700 °C for 20 min in a hydrogen atmosphere. The film was deposited on the substrate by thermal evaporation at a pressure of 10^−6^ Torr. The diffusion of Ni on Pt guarantees the absence of catalyst particles directly on the electrodes, and the growth of MWCNTs only on the Si_3_N_4_ substrate. The MWCNTs were grown by keeping the substrate at a temperature of 700 °C for 10 min in an acetylene atmosphere. In Figure 2a, a scanning electron microscopy image of the resulting MWCNT is reported and in the inset a Raman spectrum of MWCNT exhibits two main peaks attributed to the D- and G-bands. The G-band at ≈1600 cm^−1^ corresponds to the splitting of the *E*_2_*_g_* stretching mode of graphite. The intense D-band indicates the presence of defective graphitic structures or amorphous carbon [14].

Regarding the electrical measurements performed, a drain voltage was applied between the topside and backside of the electrodes (Figure 2b). The topside electrodes were both connected to ground. The investigation of the device behavior as a radiation detector was performed with continuous emitting laser diodes (LDs) at several wavelengths. The LD intensity was controlled by a low voltage power supply and measured with a power meter. Measurements were performed at room temperature, at LD powers from 0.1 to 1.0 mW with a 0.1 mW step, with a drain voltage ranging from −5 to 30 V with a step of 0.1 V, and at fixed excitation wavelengths of 378, 405, 532, 650, 685, 785, 880 and 980 nm. The current was measured with a Keithley 2635 source meter, which also provided the drain voltage. The measurement procedure was controlled by LabView routines running on a PC.

## Results and Discussion

**Figure 3:**
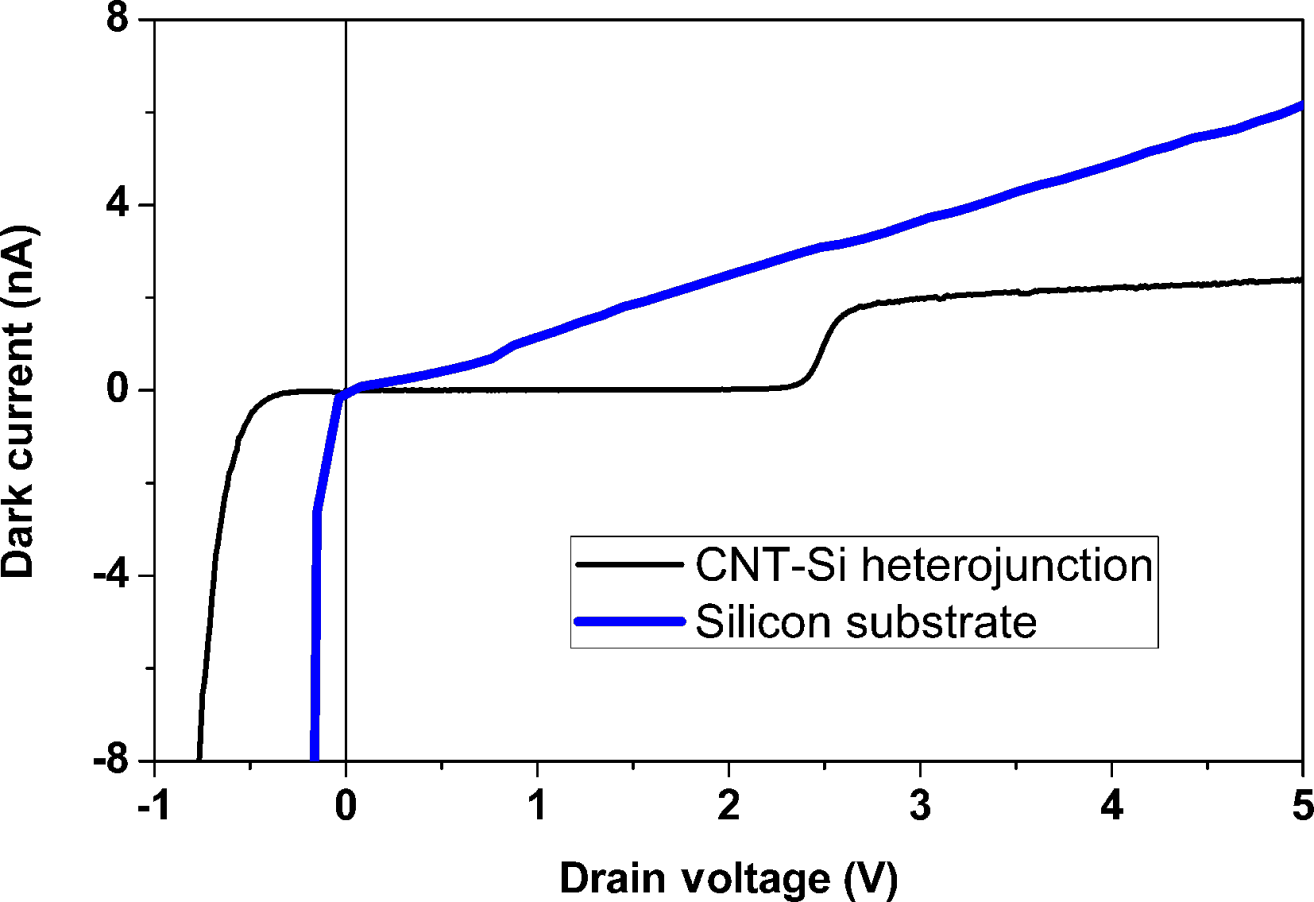
Dark current comparison of the Si substrate and the CNT–Si heterojunction.

Measurements were carried out on both the CNT–Si heterojunction and the Si substrates to compare the behavior of the pure substrate and the CNT–Si junction. Figure 3 shows the comparison between the dark currents of the bare substrate and of the CNT–Si heterojunction. The curves were obtained after stressing the junctions through different sweep voltages in the range between −30 and 30 V. Both of the devices showed a rectifying behavior after the conditioning – an indication that the conduction channels are open through the nitride layer by the voltage sweeps.

The reverse current for positive voltages, however, is very different. As the substrate shows a linear trend due to internal thermionic emission and a low shunt resistance, the CNT–Si junction exhibits a null dark current until a threshold is reached. For this device, the threshold was found at 2.4 V. Above this threshold, the current assumes a linear trend. In any case, the thermionic current through the heterojunction is less than that present in the substrate alone.

**Figure 4:**
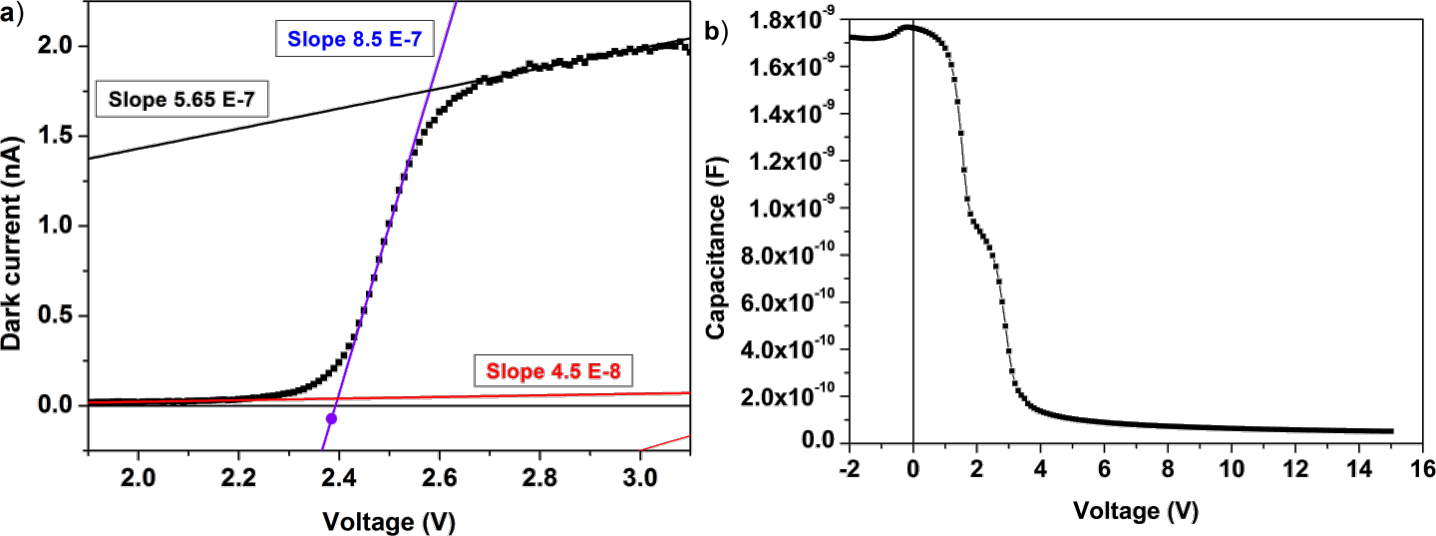
(a) Details of the dark current around the threshold voltage with a curve fit. (b) C–V plot of the heterojunction.

The detailed characteristics of the dark current around the threshold voltage are shown in Figure 4a, and Figure 4b shows the plot of the capacitance–voltage (C–V) measurement, which evidence the rapid decrease of the charge accumulation layer of the heterojunction around the threshold.

The CNT–Si junction exhibits interesting photosensitivity properties. While the substrate is light insensitive, the device with CNT deposited on the Si_3_N_4_ layer is greatly sensitive to radiation in the range from 378 to 980 nm.

**Figure 5:**
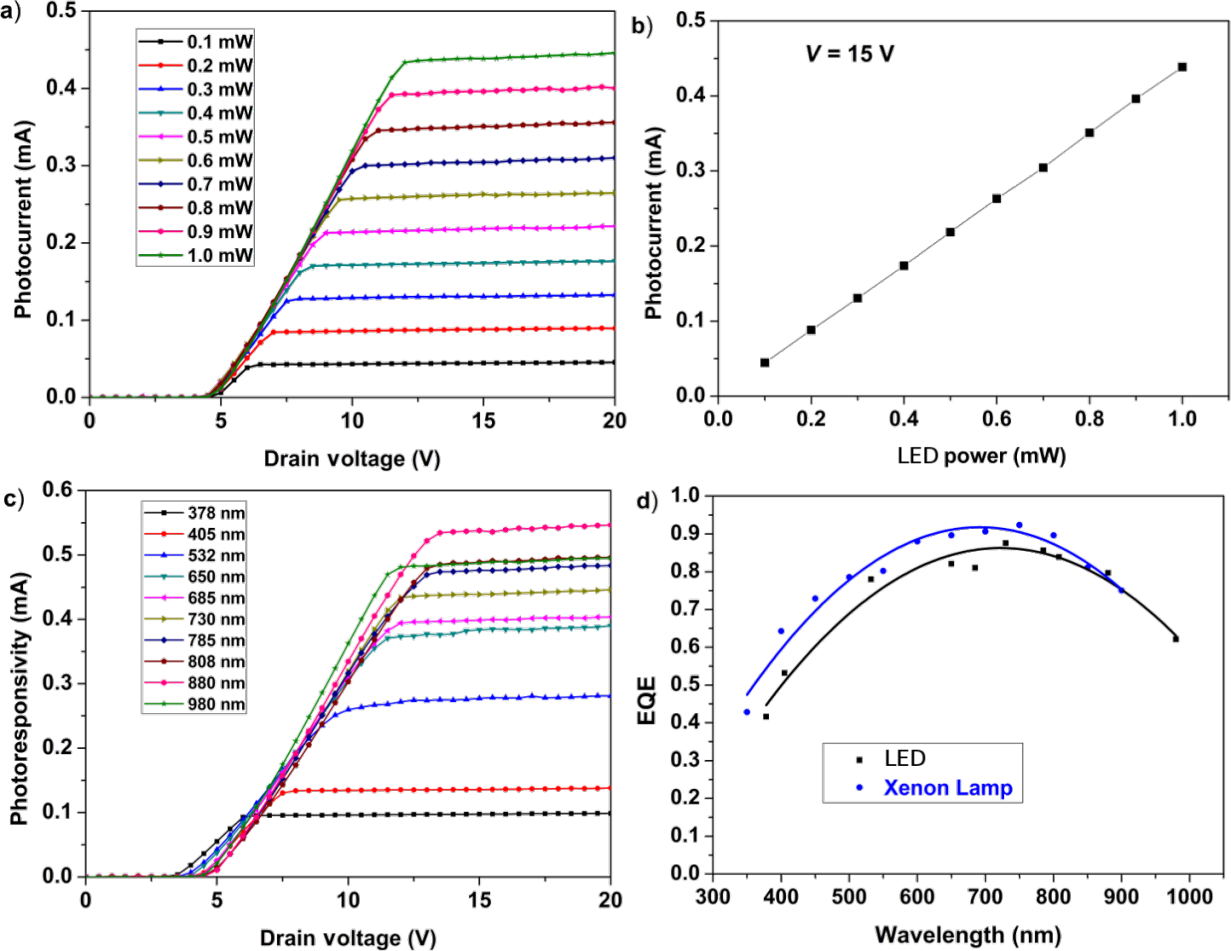
(a) Photocurrent induced by a 730 nm continuous wave, low power light source at various illumination intensities. (b) Photocurrent linearity at a drain voltage 15 V and wavelength of 730 nm. (c) Photocurrent induced at different wavelengths. (d) Comparison between the device external quantum efficiency (EQE) measured with an LD and a xenon lamp, filtered at different wavelengths.

Figure 5a reports the photocurrent measured in the configuration shown in Figure 2b. When the drain voltage exceeds the threshold voltage shown in Figure 3, the reverse photocurrent begins to grow linearly until reaching a plateau, which is constant over a large voltage range. The photocurrent depends quite linearly on the intensity of the illumination, as shown in Figure 5b. No saturation effects were observed up to tens of mW. The photodetector is sensitive to light radiation over a wide range of wavelengths.

Figure 5c shows the measured photoresponsivity (photocurrent generated by 1 mW of light intensity) for incident light of wavelengths ranging from 378 to 980 nm. When illuminated by the monochromatic intensity of a filtered xenon lamp, the external quantum efficiency (EQE) trend is similar to that of the LED illuminated experiment, as shown in Figure 5d.

It should be noted the efficiency of the detector for near-ultraviolet radiation is well above that of the Si photodetectors. This effect was observed in several similar devices as reported in [13,15–17]. However, in this report, there are some relevant, new aspects to be noted. The first one is that the EQE of the present device exhibits a maximum around 700 nm, which is at a wavelength much shorter than observed in earlier works [13]. In addition, the EQE shape is more symmetric over a large wavelength range and remains high at wavelengths from the near-UV to near-IR. The second important difference is the smaller threshold value obtained in this case. Both of these results lead to improved performance of the current device. Moreover, for these devices, we observed for the first time a non-zero current in the reverse voltage region below the 2.4 eV junction threshold under light. The shape of the current–voltage curve presents a NDR and resembles that of a resonant tunneling junction.

**Figure 6:**
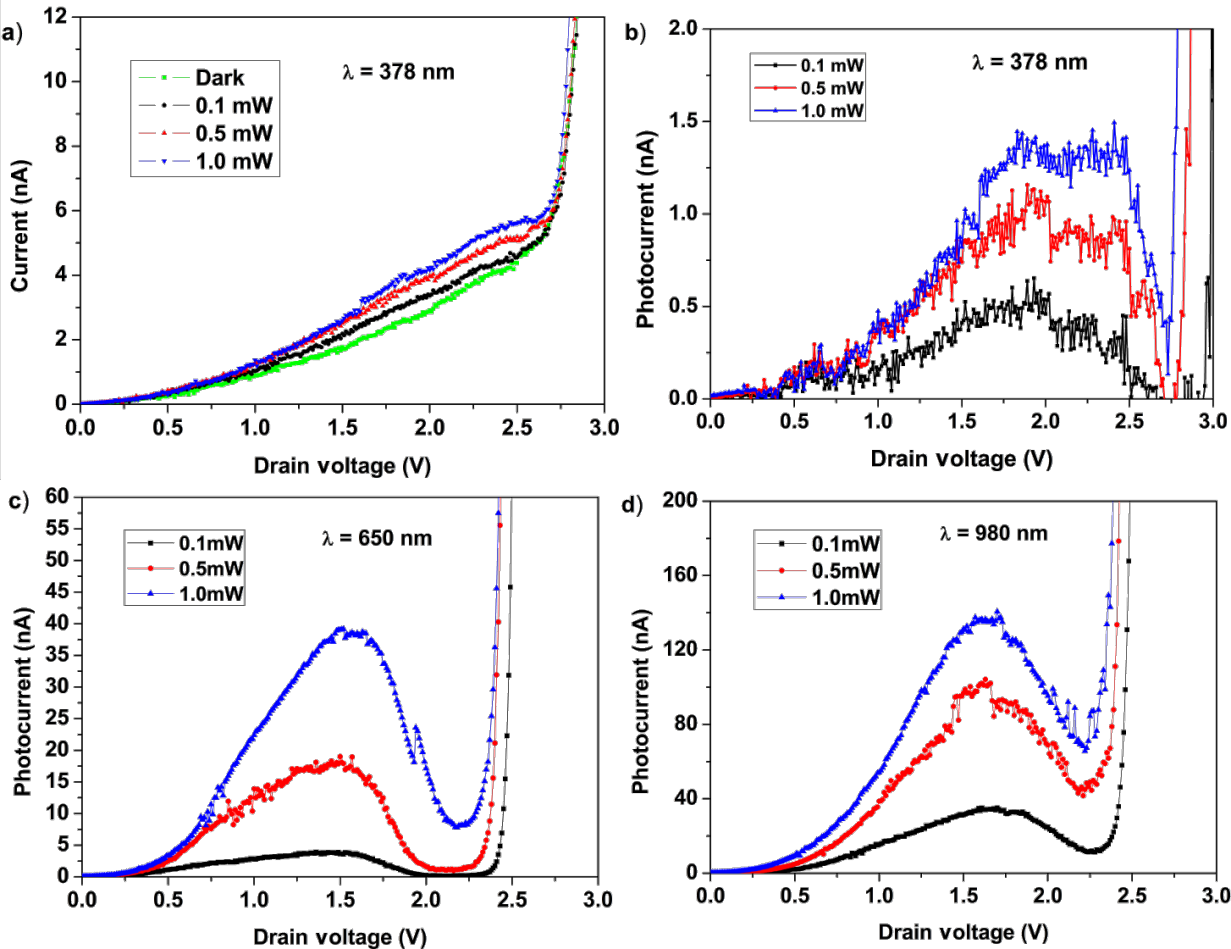
(a) Dark current and photocurrent tunneling in a CNT–Si heterojunction under 378 nm light illumination at different intensities. (b) The same as in (a) after subtracting the dark current. (c) The same as in (b) but for 650 nm and (d) 980 nm.

Figure 6b–d shows the photocurrent measured at three incident light powers (0.1, 0.5 and 1.0 mW) for three wavelengths (378, 650 and 980 nm). The drain voltage at maximum photocurrent varies weakly as a function of the wavelength of the incident radiation and is at about 1.8 V for 378 nm, 1.5 V for 650 nm and 1.7 V for 980 nm. The ratio between the peak and valley tunnel photocurrent depends on the light intensity and wavelength, as well as the NDR. The peak current is proportional to the EQE of photoconversion to any intensity and any wavelength, and is about 5 × 10^−4^ times the corresponding reverse current (at plateau) for all intensities and at all wavelengths. These effects were tested in a number of samples. Figure 5 and Figure 6 show the data obtained for two of these samples. Thus, the values reported for the ratio between the NDR peak current and the reverse current at plateau cannot be calculated given the current values reported in the two figures.

These observations clearly indicate that incident light and (as a consequence) the photogenerated charges play a fundamental role in the heterojunction behavior. The similarity in the current shape with that of a typical resonant tunneling junction suggests that a kind of electronic resonance process induced by the photogenerated charges may be present. Recently, Castrucci et al. [18] stressed that multiwall CNTs can contribute to the photocurrent because their density of states shows the same van Hove singularities as the single-walled CNTs. The excitation of electron–hole pairs is the responsible for this effect in each single wall of the multiwall CNT. In the present case, the incident light produces the sizeable absorption band observed around 1.5–2.4 eV that is a convolution of the several electronic transitions occurring in each nanotube. The contacts among the nanotubes ensure the charge transfer between the nanotubes and the observation in the I–V curve. The bell shape of the absorption band detected in the I–V spectra mimics that observed in the tunneling effect between a highly doped p–n junction; however, in our case, the physics behind this process is completely different. However, several questions are still open regarding the interpretation of the experimental data, for which we cannot exclude the presence of different mechanisms.

## Conclusion

In this paper, we report the results of a negative differential resistance behavior generated by the incident radiation, which varies as a function of wavelength and incident power intensity for a new photosensitive device consisting of MWCNTs grown at 700 °C on a Si substrate. The junction presents rectifying properties with a 2.4 V threshold to the flow of reverse current, a strong photosensitivity to light radiation at wavelengths between 378 and 980 nm, a very broad plateau extended over a large range of drain voltages, and a good linearity of the photoresponsivity versus light intensity. The conversion efficiency of light radiation to photocurrent is maximum at 730 nm, with an external quantum efficiency of ≈92%, and an EQE of ≈43% at 378 nm. No saturation phenomena were observed at high intensity, and no significant differences between the diffuse light of a xenon lamp and the directed light of LDs were observed.

The most surprising result was the observation of a remarkable photoinduced resonant tunneling-like current, which was completely absent in dark conditions, and which was absent in the substrate without CNTs. Therefore, the resonant tunnel-like current is generated only under light radiation and it is function of the wavelength as well as of the power intensity. The ratio between the resonant tunneling-like peak photocurrent and the plateau of the reverse photogenerated current was about 5 × 10^−4^ for all intensities and wavelengths. These features, which are currently still under investigation, suggest the potential use of the device for optoelectronics applications.
